# Author Correction: Microphysiological model reveals the promise of memory-like natural killer cell immunotherapy for HIV^±^ cancer

**DOI:** 10.1038/s41467-023-43057-w

**Published:** 2023-11-10

**Authors:** Jose M. Ayuso, Mehtab Farooqui, María Virumbrales-Muñoz, Katheryn Denecke, Shujah Rehman, Rebecca Schmitz, Jorge F. Guerrero, Cristina Sanchez-de-Diego, Sara Abizanda Campo, Elizabeth M. Maly, Matthew H. Forsberg, Sheena C. Kerr, Robert Striker, Nathan M. Sherer, Paul M. Harari, Christian M. Capitini, Melissa C. Skala, David J. Beebe

**Affiliations:** 1 Department of Pathology & Laboratory Medicine, University of Wisconsin School of Medicine and Public Health, Madison, WI USA; 2 Department of Dermatology, University of Wisconsin School of Medicine and Public Health, Madison, WI USA; 3 The University of Wisconsin Carbone Cancer Center, University of Wisconsin, Madison, WI USA; 4 Department of Cell and Regenerative Biology, University of Wisconsin School of Medicine and Public Health, Madison, WI USA; 5 Morgridge Institute for Research, 330 N Orchard street, Madison, WI USA; 6 Department of Biomedical Engineering, University of Wisconsin, Madison, WI USA; 7 McArdle Laboratory for Cancer Research, University of Wisconsin, Madison, USA; 8 Institute for Molecular Virology, University of Wisconsin, Madison, WI USA; 9 Department of Pediatrics, University of Wisconsin School of Medicine and Public Health, Madison, USA; 10 Department of Medicine, University of Wisconsin School of Medicine and Public Health, Madison, USA; 11 Vivent Health, Milwaukee, USA; 12 Department of Human Oncology, University of Wisconsin School of Medicine and Public Health, Madison, WI USA

**Keywords:** Immunology, Biomedical engineering, Translational immunology, Tumour immunology

Correction to: *Nature Communications* 10.1038/s41467-023-41625-8, published online 21 October 2023

In this article, the author name Paul M. Harari was incorrectly written as Paul Harari. The original article has been corrected.

