# Supplementary material for: Microphysiological model reveals the promise of memory-like natural killer cell immunotherapy for HIV± cancer
Source: Nat Commun. 2023 Oct 21;14:6681. doi: 10.1038/s41467-023-41625-8 (PMC10590421; doi:10.1038/s41467-023-41625-8)
Supplement: Supplementary file 1 — Supplementary Information [file 41467_2023_41625_MOESM1_ESM.pdf]

Microphysiological model reveals the promise of memory-like Natural Killer cell immunotherapy for HIV+ cancer

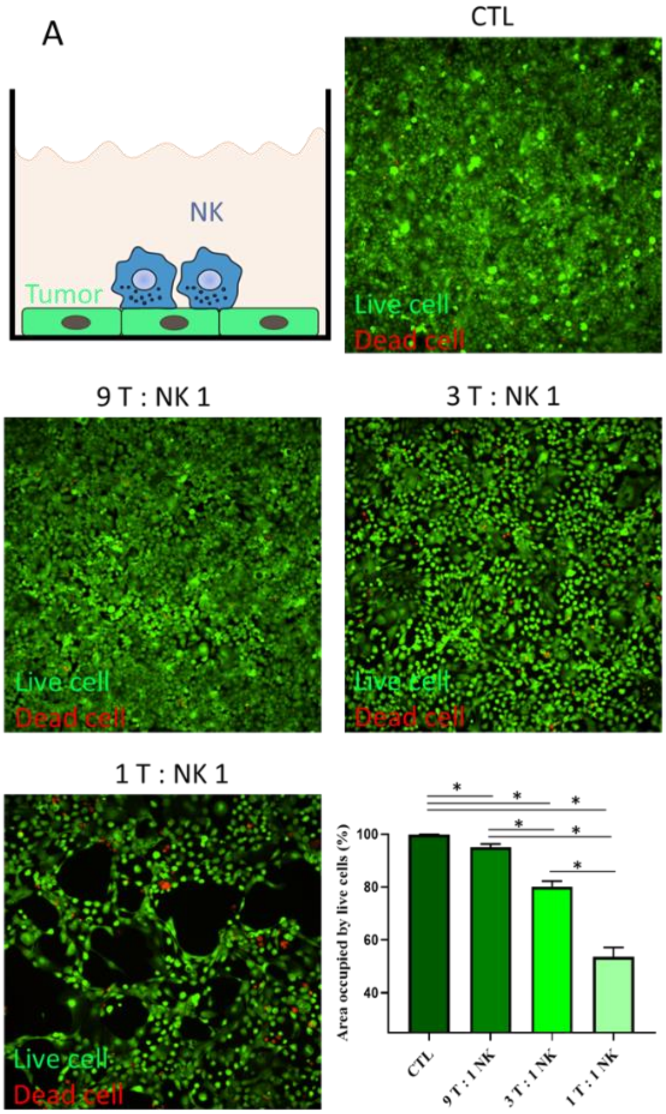

Supporting Figure 1. NK -92 cell cytotoxicity against SCC-6. NK-92 cells were seeded on top of a SCC-6 monolayer for 24 hours. NK-92 cells and dead SCC-6 cells were washed away and the reamiing attached cells were stained with CAM and PI, labelling viable and dead cells in green and red, respetcivly. Fluorescence images show a representative result. The experiments were repeated three independent times, acquiring five images per experiment. Images from the same experiment were averaged and used as an independent replicate. Graphs show average of these three independent replicates ± standard deviation. P-value was set to 0.05.

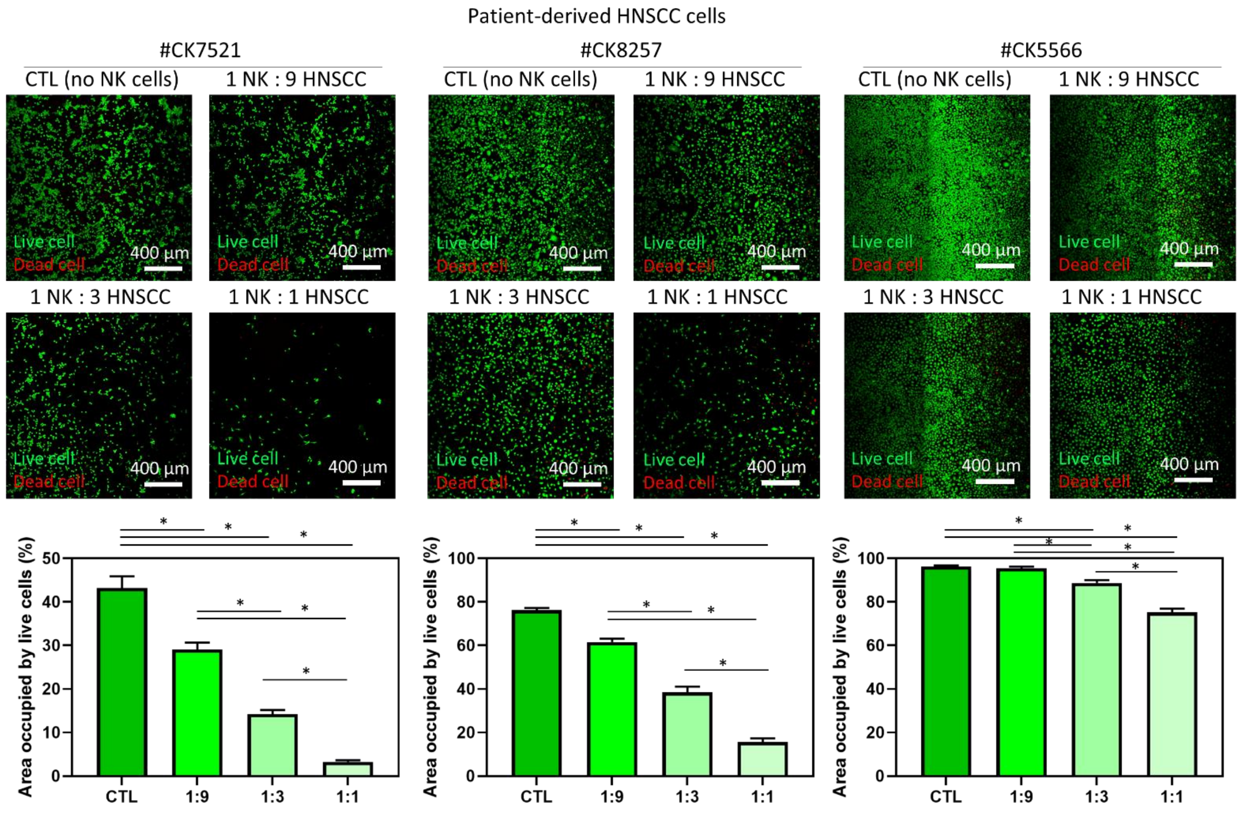

Supporting Figure 2. NK-92 cell cytotoxicity against patient-derived HNSCC cells. NK-92 cells were seeded on top of a patient-dervied HNSCC monolayer for 24 hours. NK-92 cells and dead SCC-6 cells were washed away and the reamiing attached cells were stained with CAM and PI, labelling viable and dead cells in green and red, respetcivly. Fluorescence images show a representative result. The experiments were repeated three independent times, acquiring five images per experiment. Images from the same experiment were averaged and used as an independent replicate. Graphs show average of these three independent replicates ± standard deviation. P-value was set to 0.05.

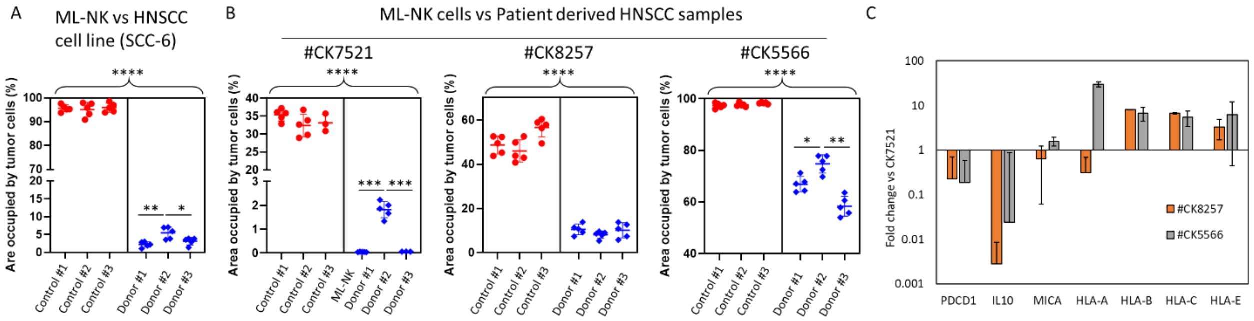

Supplementary Figure 3. Evaluaiton of ML-NK cell cytooxcity against patient-derived HNSCC samples. Primary NK cells were isolated from multiple donors and differentiated into ML-NK cells by cytokine stimulation. After 7 days, they were co-cultured with HNSCC cell lines (i.e., SCC-6) and HNSCC patient-derived samples for another 24 hours to evaluate their citotoxic potential. Graphs show average ± standard deviation. *,**, and **** indicate p-value < 0.05, 0.01, 0.001 respecticvely. Brackets indicate the the comparisson of all the control samples with the ML-NK samples done by a nested t-test. Experiments in A and B were repeated three independent times and we acquired tilescan images to cover a large area of each well-plate. C) We analyzed the expression of multiple genes associated with NK cell sensitivity and activation. The normalized fold change expression shown in the graph was calculated as follows: first we calculated the fold change of each individual gene with respect the average of five housekeeping genes for a given HNSCC sample, next we normalized that fold change with respect the fold changed obtained in CK7521 cells. Thus, this provides the fold change of each gene in CK8257 and CK5566 cells normalized to the value obtained in CK7521. Raw data can be downleaded from the NIH patient derived model repository (NIH PDMR). Graphs show average ± standard deviation. *, **, *** denote p-value<0.05, <0.01, <0.005.

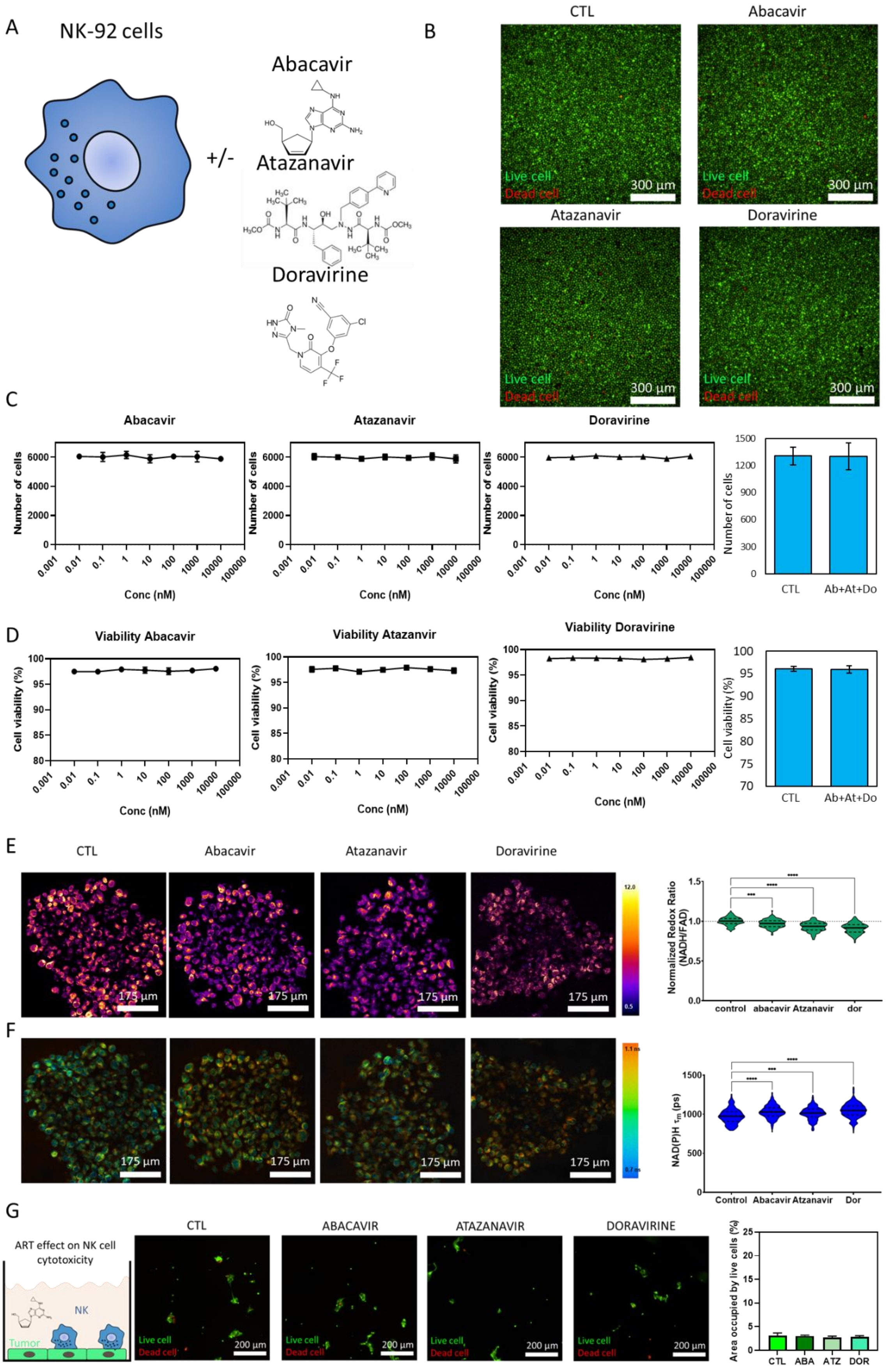
A
NK-92 cells
+/-
Abacavir
Atazanavir
Doravirine
B
CTL
Abacavir
Atazanavir
Doravirine
Live cell
Dead cell
300 µm
C
Number of cells
Conc (nM)
CTL
Ab+At+Do
D
Viability Abacavir
Viability Atazanvir
Viability Doravirine
Cell viability (%)
E
175 µm
Normalized Redox Ratio (NADH/FAD)
control
abacavir
Atzanavir
dor
F
NAD(P)H τm (ps)
Control
Abacavir
Atzanavir
Dor
G
ART effect on NK cell cytotoxicity
NK
Tumor
CTL
ABACAVIR
ATAZANAVIR
DORAVIRINE
200 µm
Area occupied by live cells (%)
CTL
ABA
ATZ
DOR

Supporting Figure 4. Evaluation of potential off-target effect of ART on NK cell biology. A) NK-92 cells were cultured in the presence of multiple anti-HIV drugs such as Abacavir (nucleoside analog reverse transcriptase inhibitor), Atazanavir (HIV protease inhibitor), and Doravirine (non-nucleoside reverse transcriptase inhibitor). B) After 7 days in culture in the presence of these compounds, NK-92 cell viability was evaluated staining live and dead NK cells with calcein AM (in green) and PI (in red). C-D) The viability analysis showed no effect on NK cell viability or proliferation at any of the concentrations tested. Culturing NK-92 cells in the presence of the three drugs showed no effect either. E-F) Optical metabolic imaging of NK-92 cells in the presence of these drugs revealed significant changes in their redox ratio as well as their NAD(P)H FLIM. G) The presence of ART drugs did not affect the cytotoxic capacity of NK-92 against SCC-6 cells. 15000 SCC-6 cells were seeded on 96 well-plates to form a monolayer. 24 hours later 15000 NK-92 cells were seeded o top and co-culture for 3 days. NK-92 cells and dead floating SCC-6 cells were removed and attached SCC-6 were stained with calcein AM (green) and PI (red). Fluorescence images show a representative result. The experiments were repeated three independent times, acquiring three images per experiment. Images from the same experiment were averaged and used as an independent replicate. Graphs show average of these three independent replicates ± standard deviation. OMI images were analyzed with cell profiler using a segmentation algorithm to identify cells, exclude the nuclear area, and quantify autofluorescence intensity. At least 100 cells were analyzed in each condition. Bar graphs show average ± standard deviation. *, **, *** denote p-value<0.05, <0.01, <0.005.

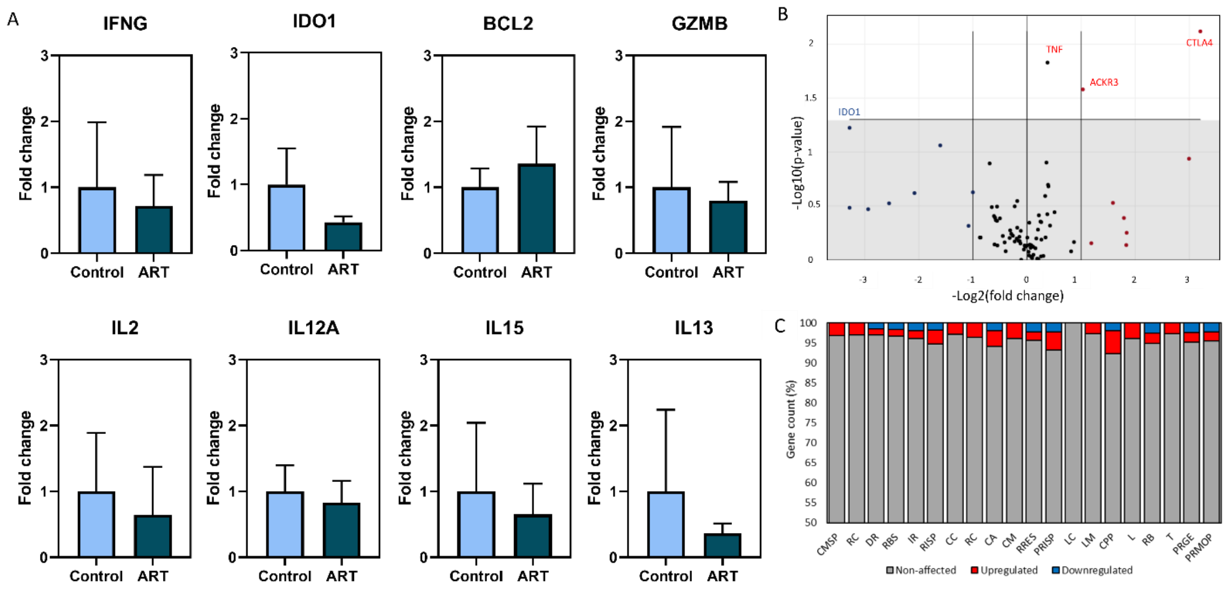

Supporting Figure 5. Evaluation of potential off-target effect of ART on ML-NK cells. ML-NK cells were cultured for 7 days in the presence of the three ART drugs (i.e., 10 µM Abacavir, Atazanavir, and Doravirine). A) Gene expression analysis in ML-NK cells in the presence of ART drugs (10 µM Abacavir, Atazanavir, and Doravirine) for 7 days. The genes showed in the bar graphs are the same ones showed in Figure 4B. ART drugs showed no significant changes in gene expression. Experiments were repeated three independent times using biological donors to derive ML-NK cells. B) Volcano plot shows gene expression in NK cells in the presence of ART drugs compared with control (i.e., DMSO). C) Gene ontology analysis showed the percentage of genes differentially expressed in the 10 biological processes analyzed in the experiment. CMSP: Cytokine mediated signaling pathway; RC: Response to cytokines; DR: Defense response; RBS: Response to biotic stimulus; IR: Inflammatory response; RISP: Regulation of immune system process; CC: Cell chemotaxis; RCh: Response to chemokine; CA: Cell activation; CM: Cell migration; RRES: Regulation of response to external stimulus; PRISP: Positive regulation of immune system process; LC: Leukocyte chemotaxis; LM: Leukocyte migration; CP: Cell population proliferation; L: locomotion; RB: Response to bacterium; T: Taxis; PRGE: Positive regulation of gene expression; PRMOP: Positive regulation of multicellular organismal process. Bar graphs show average ± standard deviation. *, **, *** denote p-value<0.05, <0.01, <0.005.

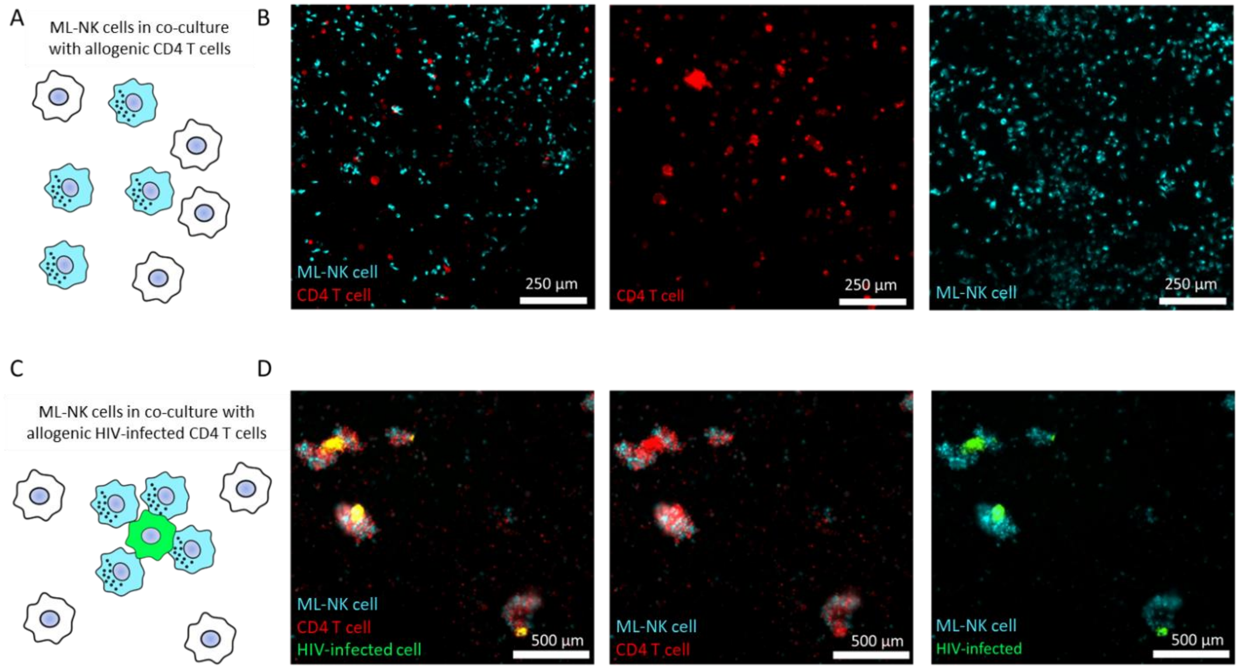

Supporting Figure 6. ML-NK cells recognize HIV infection in primary CD4 T cells. A-B) CD4 T cells (in red) were infected with a HIV fluorescent reporter (in green) by spinoculation (1200 g, 2 hours) and after 24 hours were co-cultured with allogenic ML-NK cells (in cyan) for 24 hours more. Images showed that allogenic ML-NK and CD4 T cells did not cluster together and most of the cells remained as single cells (Upper panels). C-D) On the other hand, when CD4 T cells were infected with HIV, we observed the formation of large multicellular clusters of ML-NK cells surrounding the infected T cells (lower panels). Autologous primary ML-NK and CD4 T cells (i.e., obtained from the same patient) were used in these experiments. Fluorescence images show a representative result. The experiments were repeated three independent times, acquiring three images per experiment. Images from the same experiment were averaged and used as an independent replicate. Graphs show average of these three independent replicates ± standard deviation.

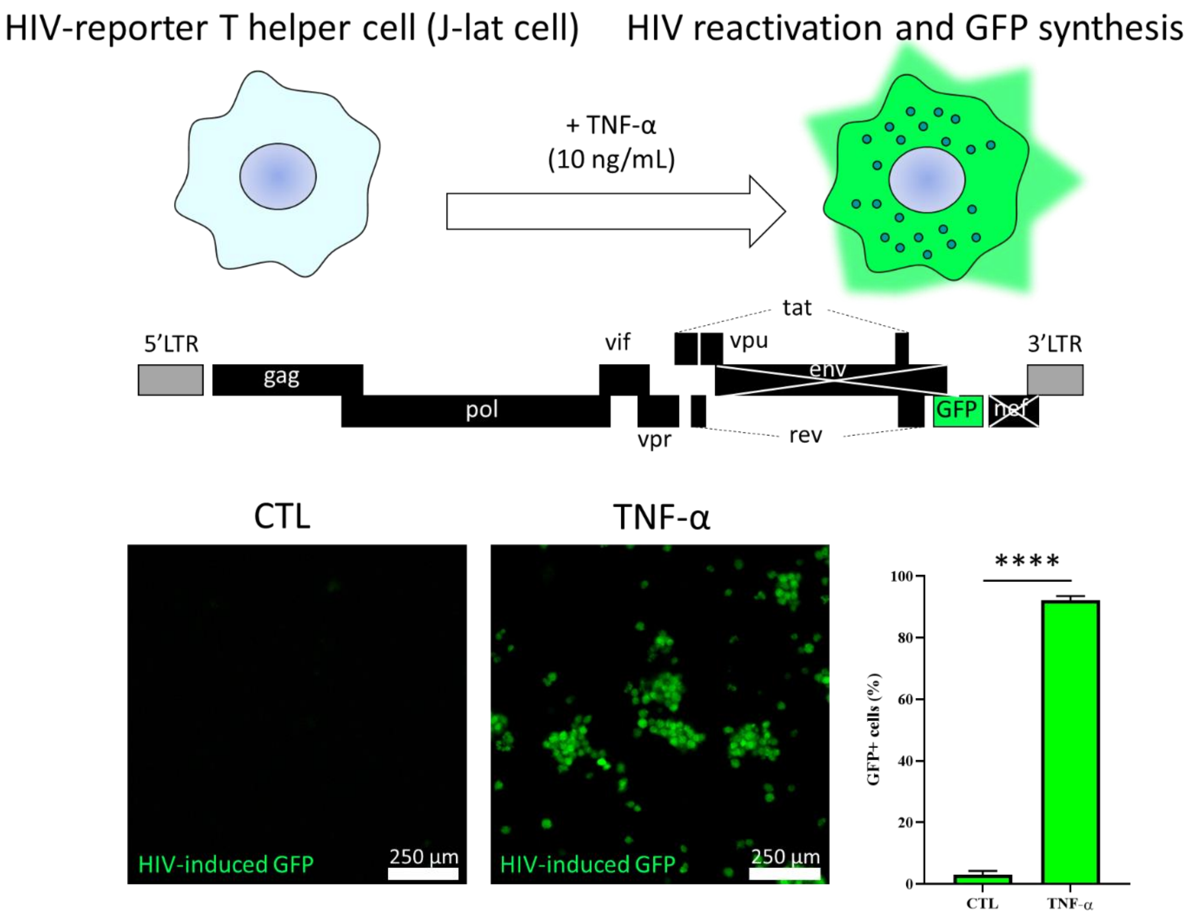

Supporting Figure 7. HIV-1 expression induction. J-lat cells are CD4 T cells that carry a dormant HIV-1 genome integrated into their DNA. The integrated HIV-1 has been modified to prevent the generation of competent progeny for safety reasons. TNF-α exposure induces HIV-1 reactivation, leading to GFP synthesis. Images show HIV-1 reactivation, detected by GFP expression in J-lat cells. Fluorescence images show a representative result. The experiments were repeated three independent times, acquiring five images per experiment. Images from the same experiment were averaged and used as an independent replicate. Graphs show average of these three independent replicates ± standard deviation. P-value was set to 0.05.

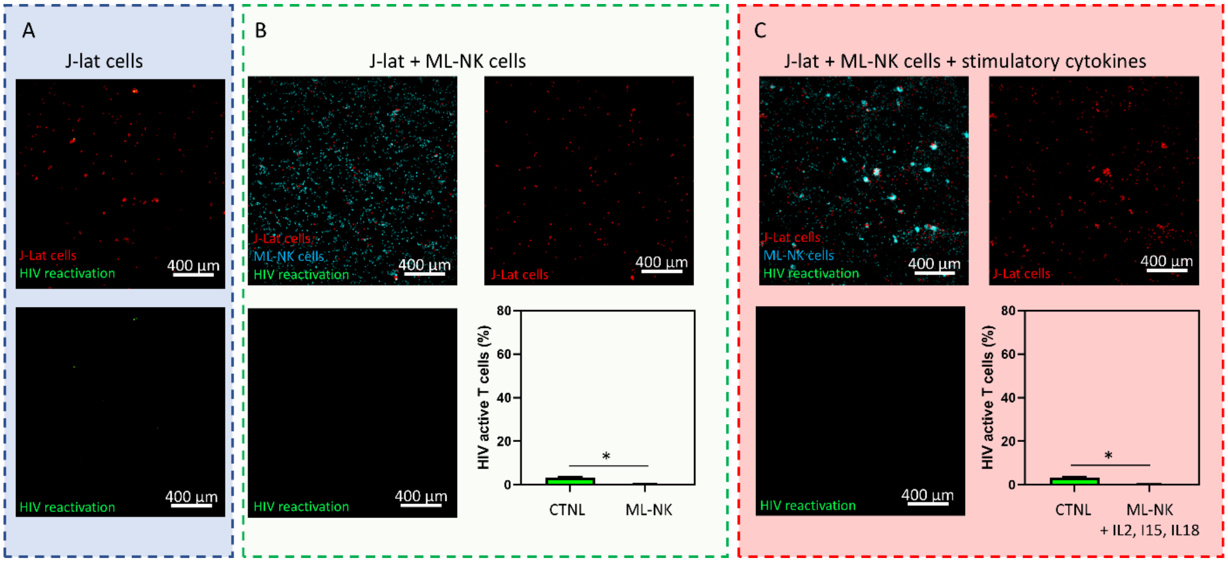

Supporting Figure 8. ML-NK cells do not reactivate dormant HIV-1 in T cells (J-lat cells). A) HIV-1 expression in J-lat cells is low in resting conditions. B) J-lat cells were co-cultured with ML-NK cells, showing a decrease of the basal HIV-1 expression in resting J-lat cells. C) J-lat cells were co-culture with ML-NK cells in the presence of the stimulatory cytokine cocktail to generate ML-NK cells (IL-12,IL-15, IL-18) to evaluate potential off-target reactivation of HIV-1. The results showed that the cytokine cocktail did not lead to HIV-1 reactivation. Fluorescence images show a representative result. The experiments were repeated three independent times, acquiring five images per experiment. Images from the same experiment were averaged and used as an independent replicate. Graphs show average of these three independent replicates ± standard deviation. P-value was set to 0.05.

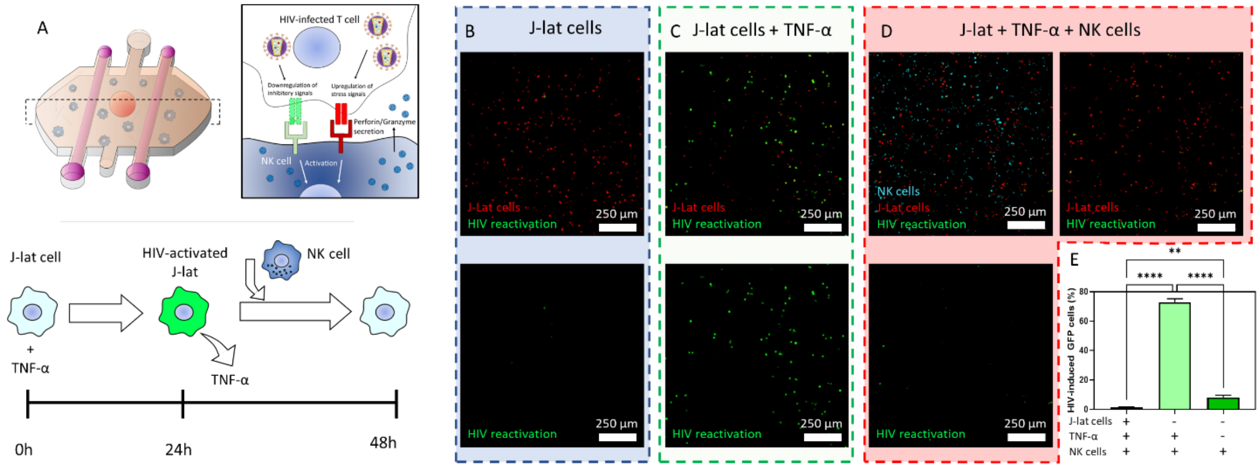

Supporting Figure 9. NK-92 cells decrease HIV-1 reactivation in J-lat cells. A) Schematic depicting the experimental timeline to evaluate the effect of NK-92 on HIV gene expression**.** B-C) HIV-1 expression (shown in green) was induced by exposing J-lat cells (shown in red) to TNF-α for 24 hours. D) After TNF-α exposure, J-lat cells were co-cultured by 24 hours in the presence of NK-92 cells (shown in cyan). E) Graph shows the percentage of J-lat cells that express HIV proteins under the experimental conditions shown in the microscopy images. Fluorescence microscopy images showed a decrease in HIV gene expression observed by a decrease in the green signal. Fluorescence images show a representative result. The experiments were repeated three independent times, acquiring five images per experiment. Images from the same experiment were averaged and used as an independent replicate. Graphs show average of these three independent replicates ± standard deviation. P-value was set to 0.05.

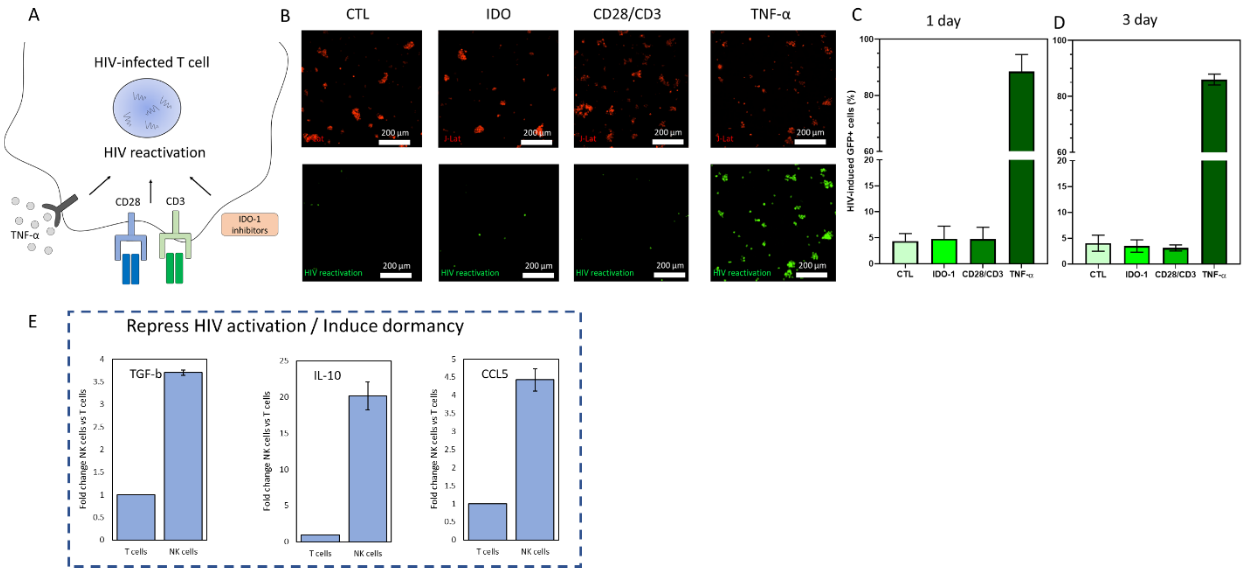

Supporting Figure 10. Modulation of latent HIV-1 expression by immunomodulatory agents and NK-92 cells. A) We evaluated potential HIV-1 reactivation in J-lat cells exposed to the tumor spheroid or compounds used to stimulate the immune response during immunotherapy treatments. B-D) J-lat cells stimulated with anti-CD3/anti-CD28 beads or IDO-1 inhibitors showed no HIV-1 activation. The presence of tumor cells led to no changes in HIV-1 reactivation after 1 or 3 days in culture. Exposure to TNF-α is shown as a positive control. E) Gene expression analysis showed NK cells expressed higher levels of several genes associated with HIV-1 dormancy and repression. Fluorescence images show a representative result. The experiments were repeated three independent times, acquiring five images per experiment. Images from the same experiment were averaged and used as an independent replicate. Graphs show average of these three independent replicates ± standard deviation. P-value was set to 0.05.

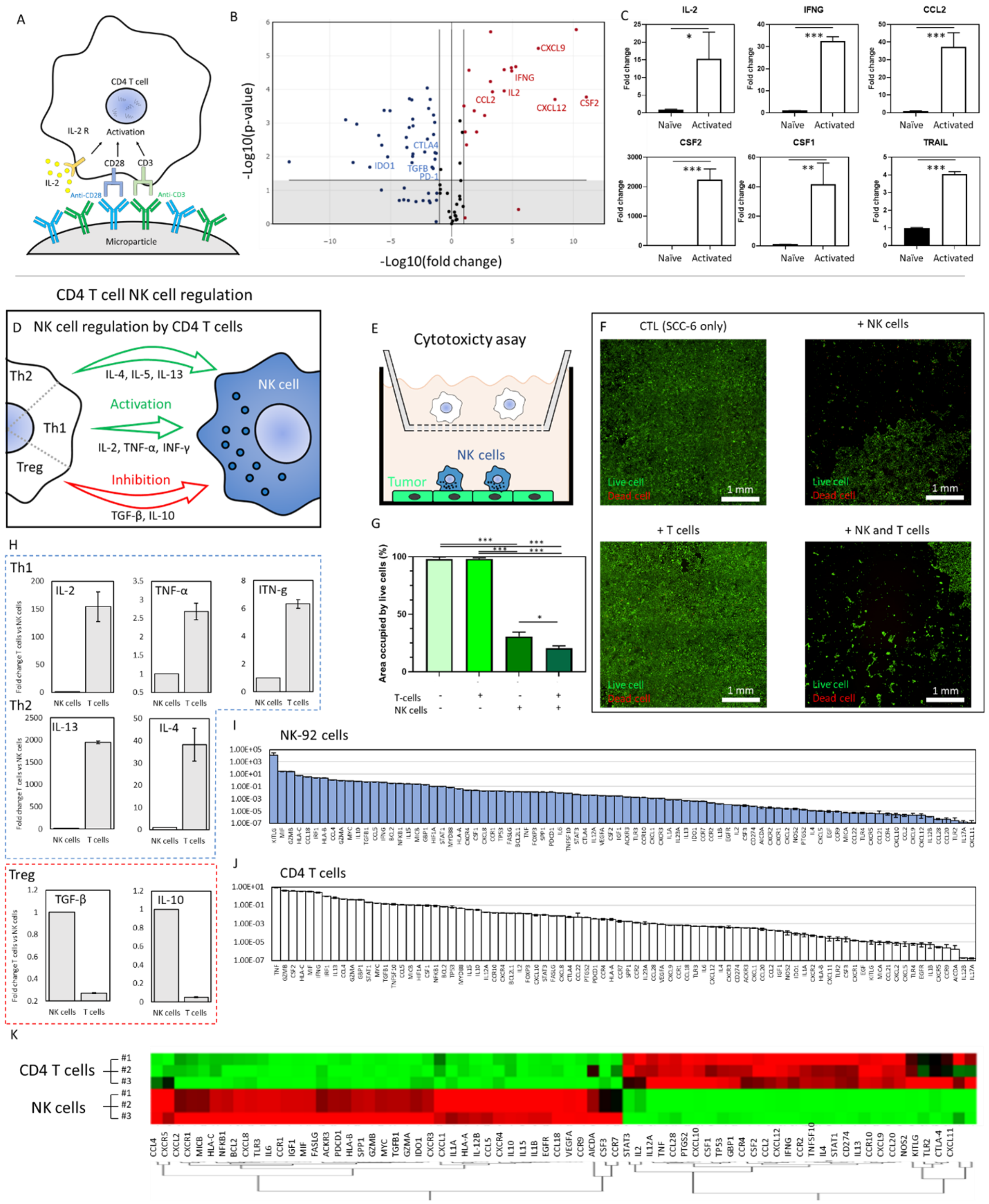

Supporting Figure 11. The presence of primary CD4 T cells increased NK-92 cell cytotoxicity. A) Primary CD4 T cells were activated by CD3 and CD28 stimulation. B) Volcano plot showing gene expression changes in activated CD4 T cells compared with resting CD4 T cells. C) CD4 T cell activation led significant changes in gene expression, including overexpression of multiple cytokines and chemokines involved in immune activation and immune response. D-E) NK-92 cells were co-cultured on top of a HNSCC SCC-6 cell monolayer at 1:1 ratio in transwell plates. Activated primary CD4 T cells were added to the insert at 1:1 ratio. F-G) After 3 days in culture, CD4 T and NK-92cells, as well as dead SCC-6 cells, were removed. Viable SCC-6 cells were stained with calcein AM (in green). CD4 T cells showed no effect on the area occupied by tumor cells,

whereas NK-92 cells reduced the tumor area >70%. The co-culture of NK-92 with CD4 T cells led to >80% reduction of the tumor area, demonstrating the positive role of CD4 T cells on NK-92 cytotoxicity. H) Gene expression analysis revealed activated CD4 T cells expressed higher levels of Th-1 and Th2-associated pro-survival and activating cytokines compared with NK-92 cells. On the other hand, Treg-associated immunosuppressive cytokines were downregulated in activated CD4 T cells compared with NK-92 cells. I-J) Waterfall plot showing gene expression in NK-92 (in blue) and activated CD4 T cells (in white). K) Non-supervised clustergram showing genes that were differentially expressed in NK-92 vs activated CD4 T cells. Fluorescence images show a representative result. The experiments were repeated three independent times, acquiring five images per experiment. Images from the same experiment were averaged and used as an independent replicate. Graphs show average of these three independent replicates ± standard deviation. P-value was set to 0.05. Bar graphs show average ± standard deviation. *, **, *** denote p-value<0.05, <0.01, <0.005.

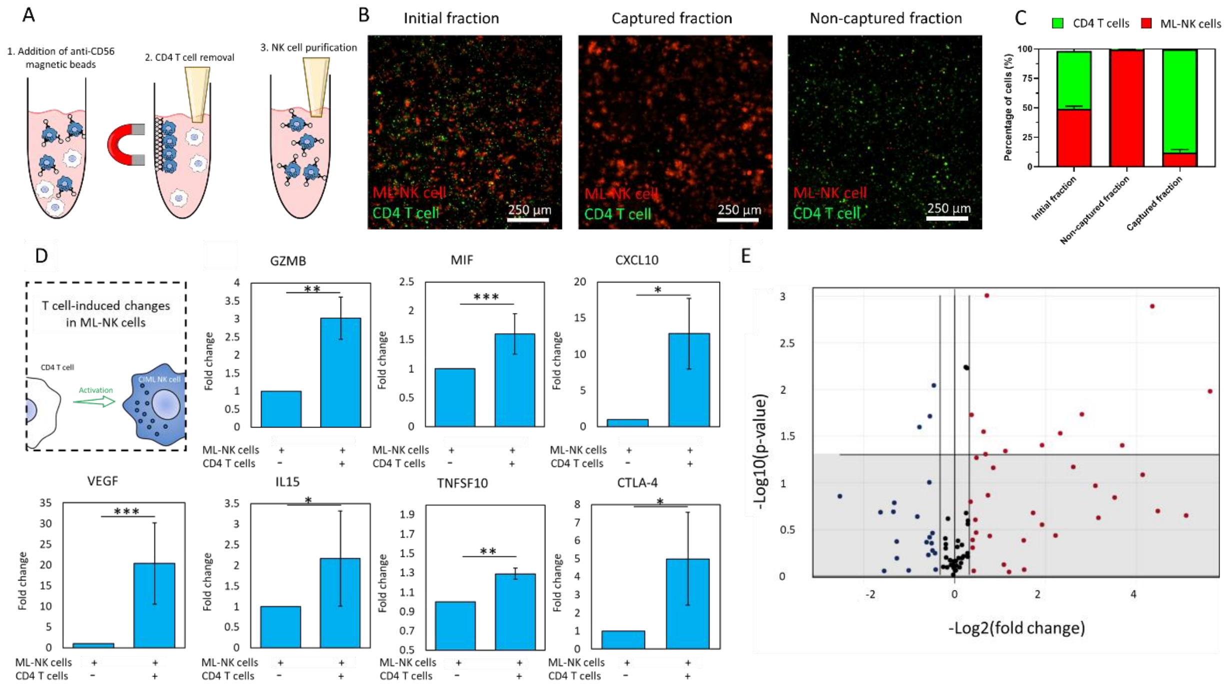

Supporting Figure 12. CD4 T cells induced gene changes in ML-NK cells. A) Schemes describing the isolation magnetic bead-based isolation protocol. B) Microscopy images show CD4 T cells and ML-NK cells in green and red respectively. C) Graph shows the percentage of CD4 T cells and ML-NK cells in the different fractions. D) We isolated allogenic NK and CD4 T cells. Naïve NK cells were differentiated into ML-NK cells following the cytokine stimulation described, whereas CD4 T cells were activated by IL-2 and CD3/CD28 stimulation. Next, ML-NK and activated CD4 T cells were cocultured together for 3 days before isolating ML-NK cells for PCR analysis. Bar graphs showing genes with significantly different expression due to the presence of CD4 T cells. E) Volcano plot summarizing the gene expression changes in ML-NK cells caused by CD4 T cells. Experiments were repeated three independent times, using several ML-NK cells donors. Fluorescence images show a representative result. Graphs show average ± standard deviation. P-value was set to 0.05.
